# Detection and investigation of temporal clusters of congenital anomaly in Europe: seven years of experience of the EUROCAT surveillance system

**DOI:** 10.1007/s10654-015-0012-y

**Published:** 2015-04-04

**Authors:** Helen Dolk, Maria Loane, Conor Teljeur, James Densem, Ruth Greenlees, Nichola McCullough, Joan Morris, Vera Nelen, Fabrizio Bianchi, Alan Kelly

**Affiliations:** EUROCAT Central Registry, WHO Collaborating Centre for Surveillance of Congenital Anomalies, Institute for Nursing and Health Research, Ulster University, Shore Rd, Newtownabbey, BT370QB UK; Department Public Health and Primary Care, Trinity College Dublin, Dublin, Ireland; Biomedical Computing Ltd, St. Leonards-on-Sea, UK; Wolfson Institute of Preventive Medicine, Queen Mary University of London, London, UK; Provinciaal Instituut voor Hygiëne, Antwerp, Belgium; Unit of Epidemiology, CNR Institute of Clinical Physiology, Pisa, Italy

**Keywords:** Congenital anomalies, Surveillance, Clusters

## Abstract

Detection and investigation of congenital anomaly clusters is one part of surveillance to detect new or changing teratogenic exposures in the population. The EUROCAT (European Surveillance of Congenital Anomalies) cluster monitoring system and results are described here. Monitoring was conducted annually from 2007 to 2013 for 18 registries covering an annual birth population up to 0.5 million births. For each registry and 72 anomaly subgroups, the scan “moving window” technique was used to detect clusters in time occurring within the last 2 years based on estimated date of conception. Registries conducted preliminary investigations using a standardised protocol to determine whether there was cause for concern, and expert review was used at key points. 165 clusters were detected, a rate of 3.4 % of all 4823 cluster tests performed over 7 years, more than expected by chance. Preliminary investigations of 126 new clusters confirmed that 35 % were an unusual aggregation of cases, while 56 % were explained by data quality or diagnostic issues, and 9 % were not investigated. For confirmed clusters, the registries’ course of action was continuing monitoring. Three confirmed clusters continued to grow in size for a limited period in subsequent monitoring. This system is best suited to early detection of exposures which are sudden, widespread and/or highly teratogenic, and was reassuring in demonstrating an absence of a sustained exposure of this type. Such proactive monitoring can be run efficiently without overwhelming the surveillance system with false positives, and serves an additional purpose of data quality control.

## Introduction

The thalidomide epidemic in the early 1960s, when more than 10,000 babies worldwide were born with major congenital anomalies due to early pregnancy exposure to the medication thalidomide [1], was a seminal event for both congenital anomaly (CA) surveillance and pharmacovigilance. European Surveillance of Congenital Anomalies (EUROCAT) was set up in its wake, with a focus on the early detection of any new epidemic of CA related to teratogenic exposure [2]. Now, more than 50 years after thalidomide, EUROCAT covers nearly one-third of the 5 million births annually in the European Union (EU). The interest of surveillance has widened to other environmental exposures beyond medications, in particular environmental pollutants. Electronic data transfer and linkage, greater computing power, and new statistical methodology have presented new opportunities for the conduct of surveillance.

At the core of CA surveillance is the regular and systematic detection of increasing trends and clusters in time, to detect potential concerns where there is no prior hypothesis about the exposure, an activity EUROCAT terms “Statistical Monitoring” [3, 4]. This may provide the first clue to a teratogenic exposure, eventually leading to a preventive intervention which can stop the increase in frequency continuing or reoccurring. Nevertheless, outside of applications in infectious disease control and biosurveillance, cluster detection has been a controversial area in public health practice [4–6], with concerns that it could overwhelm a public health system with chance clusters [5, 6], that it is difficult to distinguish clusters with a common causal agent from chance clusters or find that common cause [7], that routine detection systems are too slow to detect increases [5], and that cluster detection methods are poorly characterised in terms of their power to detect clusters of interest [8].

In this paper, we present the EUROCAT approach to the detection and investigation of temporal clusters, and the results to date. The routine assessment of trends which also forms part of EUROCAT statistical monitoring has been described previously [3], and the statistical methods for cluster detection are presented in full in an accompanying paper [9].

## Methods

### EUROCAT registries and database

EUROCAT registries are population-based, and cover livebirths, fetal deaths from 20 weeks gestational age (GA), and terminations of pregnancy for fetal anomaly. Registries use multiple sources of case ascertainment [10], and data quality is described and monitored by a series of data quality indicators [11].

There are 31 full and 6 associate member EUROCAT registries currently, covering a population of 1.6 million births per year. Full member registry populations range from a few small registries with <10,000 births per year, to the majority of registries in the range 10–50,000 births, to two registries with 75–90,000 annual births [10]. Only registries with stable birth populations (varying <10 % per year), and which transmit data before the set deadline, are eligible to participate in monitoring [9, 12]. The number of registries eligible each year is shown in Table 1. Up to 18 registries were eligible: Antwerp, Hainaut, (BE); Odense, (DK); Isle de La Reunion, Paris, (FR); Dublin, South East Ireland, (IE); Emilia Romagna, Tuscany, (IT); Northern Netherlands, (NL); Vaud (CH); Ukraine (UA); East Midlands and South Yorkshire, North England, South West England, Thames Valley, Wales, Wessex (UK) EUROCAT has a Central Registry which holds a database of anonymised individual records of cases of CA for full members, and aggregate data for associate members. The dataset of individual records used for statistical monitoring includes date of birth (DOB), gestational age (GA, in completed weeks), type of birth (live, still, termination of pregnancy) and up to nine malformations and syndromes coded to ICD10-BPA [13].

**Table 1:** Cluster detection for the period 2007–2013 by year: no. EUROCAT registries included in monitoring and their population coverage, number of tests conducted and clusters detected, and results of cluster investigations

	2007	2008	2009	2010	2011	2012	2013	Total 2007–2013
Results of cluster detection								
Registries (n)	10	11	10	16	16	17	18	–
Annual population covered (no. births)	234,317	226,011	272,975	390,510	401,282	473,675	505,250	–
Cases of Congenital Anomaly (n)	11,891	10,687	12,048	16,654	18,275	22,152	23,619	–
Congenital anomaly subgroups (n)	75	75	75	76	76	73	72	–
Tests performed (n)	482	500	487	766	805	882	901	4823
Cluster detection rate (%)	2.90	3.40	4.72	3.92	2.48	3.06	3.77	3.42
Total clusters detected (n)	14	17	23	30	20	27	34	165
New^a^ clusters (n)	12	15	18	21	16	24	20	126
Old^a^ clusters (n)	2	2	4	4	3	3	7	25
Continuing^a^ Cluster (n)	0	0	1	5	1	0	7	14
Investigation results (new clusters only)								
Cluster confirmed, no explanation	2	9	6	7	2	11	7	44 (34.9 %)
Cluster due to data quality issues	3	3	5	6	2	6	10	34 (27.8 %)
Cluster due to increasing prenatal diagnosis	0	0	2	1	7	0	0	10 (7.9 %)
Heterogeneous anomalies in cluster	4	3	2	3	5	6	3	26 (20.6 %)
No investigation	3	0	3	4	0	1	0	11 (8.7 %)

^a^New clusters are those not detected in the previous year’s monitoring, old clusters are clusters detected in the previous year’s monitoring which have not grown in size, continuing clusters are those clusters detected in the previous year’s monitoring which have since continued and increased in size

### Definition of “cluster” and time and spatial dimensions

EUROCAT defines a temporal cluster as an unusual aggregation of cases in time. This definition does not carry any judgement as to whether the cluster may or may not be a chance phenomenon. For cluster detection, an arbitrary threshold of “unusual” is used (see below).

For Central Registry analysis, the spatial dimension during the study period was operationalized as registry: which may be a region or the whole country. Since the study period for this paper, additional country level analyses have also been performed combining more than one registry in the same country. During preliminary investigation of time clusters, the spatial dimensions within the registry area are further investigated, and local registries can run the cluster detection software at subregional level.

The date of last menstrual period (LMP) calculated from DOB and GA is used as a proxy for “date of conception” and is estimated for each CA case (formally, 2 weeks should be added to LMP to arrive at estimated date of conception, but we retain an LMP-based analysis). The switch from DOB-based analysis to date of conception-based analysis was made after many clusters by DOB were found not to be clustered when considered by estimated date of conception, particularly for anomalies where terminations of pregnancy are frequent. Since we are mainly looking for signs of early pregnancy exposure, acting during specific developmental windows, date of conception is the most relevant reference point. A specific protocol deals with missing GA, including reverting to DOB-based analysis if it is missing for more than 10 % of cases [12].

The most recent 5 years of data (by year of birth) are included in the cluster analysis, and the resulting conception period is truncated to run from 1 January of the first birth year (year X − 4), to 31 March of the most recent birth year (year X) of the monitoring period. The truncation to March 31 means that all conceptions in the most recent year of birth will have resulted in a delivery or termination of pregnancy within that year. Only clusters wholly or partially occurring within the most recent 2 years (conceptions 1 April year X − 2 to 31 March year X) form part of the statistical output. Output is limited to clusters of up to 18 months duration [9, 12].

### Statistical method and software for cluster detection

As described in full elsewhere [9, 12], the Scan (or “moving window”) method [14, 15] runs a window of from 5 to (n − 2) cases, where n is the total number of cases, across the time period of analysis, and, by comparison with simulated data, detects sequences of cases that occur in a shorter time period than would be expected by chance (*p* < 0.05). Clusters of <5 cases are not sought because experience shows that clusters of <5 cases rarely result in a satisfactory investigation outcome [7, 16], and these were not foreseen in the published method [14, 15]. This method is self-adjusting for multiple testing across window sizes, but not across different registries or CA subgroups.

A number of criteria drove the choice of statistical method for cluster detection: (a) incorporating adjustment for multiple testing (b) appropriate for small numbers of cases as CA are rare (c) not requiring the prior setting of a “baseline” or expected rate which previous experience had shown slows down the system due to the need for frequent re-agreement of baselines (d) not requiring population births data (temporal resolution below yearly birth numbers is not universally or immediately available) (e) possible to program in the EUROCAT Data Management Programme (EDMP) [9, 13] with a simple interface for use by non-statisticians and (f) relatively intuitive output for communication with public health professionals and managers. Prospective scan methods have been advocated [17], but do not suit our system as we update data from previous years and only perform monitoring centrally once per year after data transmission.

**Fig. 1:**
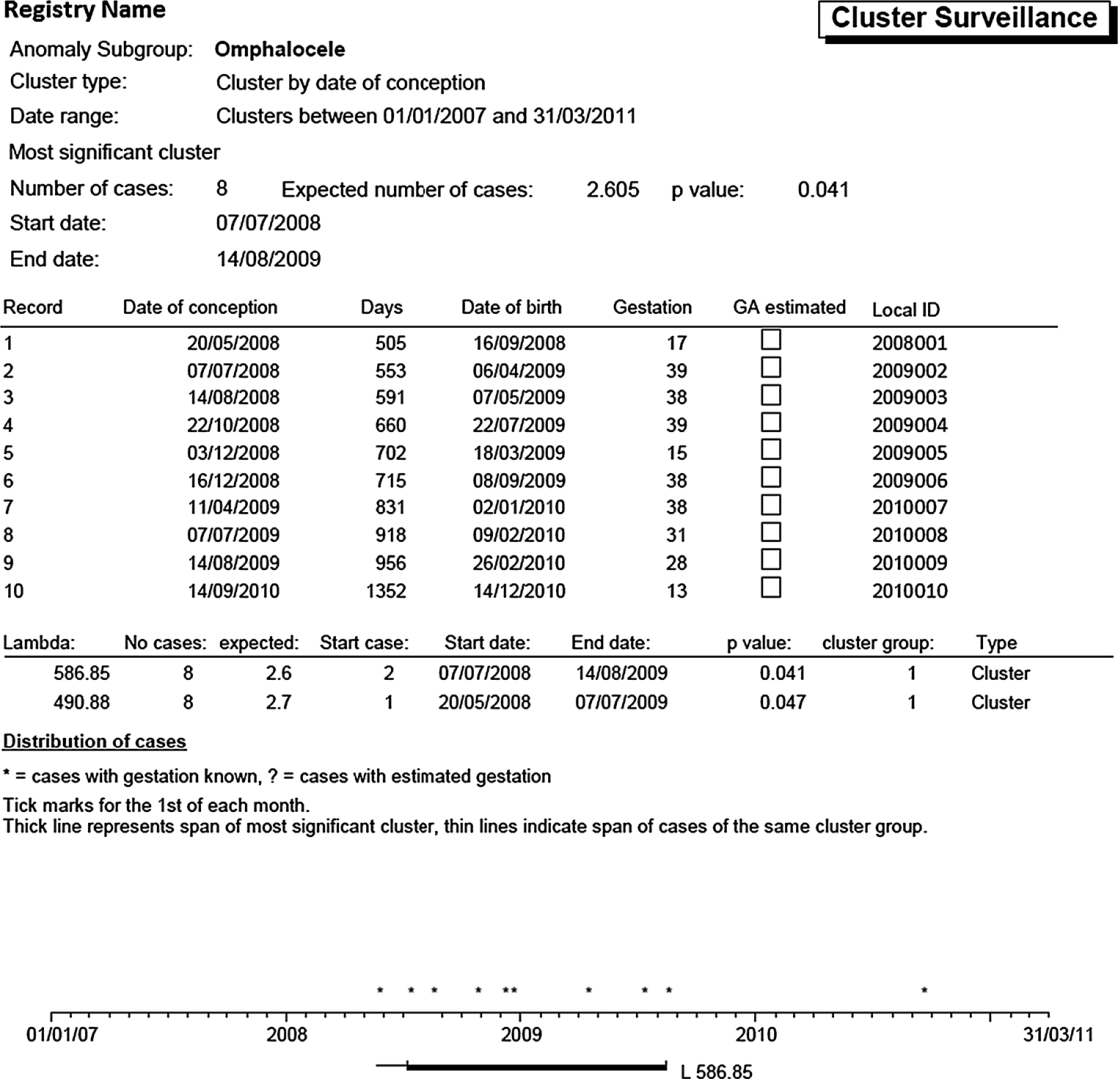
An example of the EDMP statistical monitoring output showing a cluster

The cluster detection software is integrated within the EUROCAT Central Database (ECD) software, and the EDMP used by member registries, and was refined over the years. Cluster detection output [12, 18] includes a European summary (from ECD), summaries for individual registries, and outputs detailing each specific cluster. Cluster output (Fig. 1) includes a 5 year timeline and details of the cluster with number of cases, start and end date of estimated conception, “expected” number of cases during the time period of the cluster (i.e. the average no. cases per day multiplied by the number of days), lambda (the scan statistics), and probability. Several clusters may be detected with a probability of <0.05 which overlap in time, and these are put into cluster groups [9].

### Which CA subgroups are monitored?

EUROCAT uses 89 standard binary CA subgroups, automatically assigned by the ECD/EDMP from ICD10-BPA codes [13], of which 72 are monitored for clusters [12], selected by the EUROCAT Coding and Classification Committee. Subgroups are organised at several hierarchical levels (e.g. Spina Bifida within Neural Tube Defects, or Down Syndrome within Chromosomal). Large heterogeneous subgroups (e.g. nervous system, cardiac, digestive system) are not monitored. Cases with chromosomal or other genetic syndromes are monitored separately from “non-genetic” cases. Within those categories, a case can be counted in multiple subgroups, but only once in each subgroup (e.g. a multiply malformed case of spina bifida and omphalocele is counted once for spina bifida, once for omphalocele, and once for all non-genetic).

### Preliminary investigation protocol

**Fig. 2:**
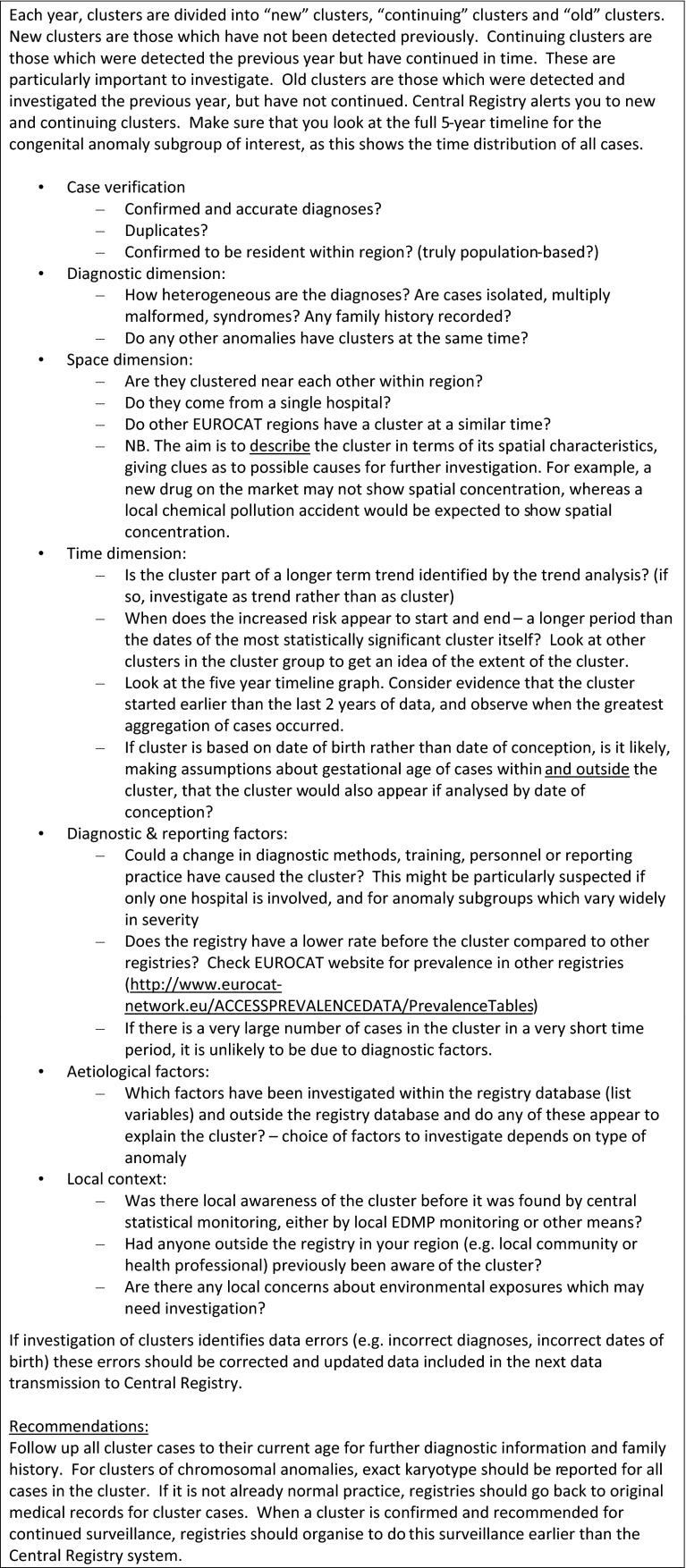
Guidelines for the preliminary investigation of clusters from EUROCAT statistical monitoring Protocol [12]

Registries conduct “Preliminary Investigations” of the clusters identified using data available in their registries, and where necessary consulting medical records or speaking with clinicians regarding changes in diagnostic methods. The protocol for preliminary investigation (Fig. 2) emphasises exploring the temporal, spatial and diagnostic dimensions of the cluster, rather than taking too arbitrary an approach to its boundaries. The first step is to verify the cases included, making sure that anomalies have been correctly coded, and that there are no hidden duplicates. If the cases are verified, the registry investigates potential changes in diagnostic or ascertainment methods as an underlying explanation. Registries then look in their records at recorded exposures e.g. medication, maternal illness, assisted reproduction, unusual exposures, and demographic characteristics such as age, ethnicity that might help seek a time-related common cause.

Registries are asked to consider the cluster detected in the light of any significant trend signalled over the same time period, and investigate as a trend rather than cluster if appropriate. They are alerted to the most significant cluster, but asked to consider the full 5-year timeline and evidence of overlapping clusters in their investigation and interpretation.

At the end of the preliminary investigation, the registry decides whether the cluster can be ascribed to an artefact of diagnostic or registry practice or data quality, or requires further monitoring or further public health investigation. We consider a cluster “confirmed” if, after preliminary investigation, the aggregation of cases remains unusual. The preliminary investigation describes the cluster (e.g. in relation to spatial aggregation within registry area) to facilitate identifying hypotheses for further investigation if appropriate, but does not conclude on or assess causality.

### Timeliness and process

Registries are included in statistical monitoring if they transmit their data for year X by February of year X + 2 (e.g. data for births in 2011 were transmitted by February 2013). This allows for postneonatal diagnosis, and for a good level of completion of case finding and data cleaning. Central Registry confirms data with member registries, runs validation checks and then runs statistical monitoring in early April.

The statistical monitoring output is assessed by a committee comprising epidemiologists, clinical geneticists, clinicians and a statistician, and including the EUROCAT Steering Committee, in mid April. The output along with their advice and a response template is sent to registries, who present their results at the Annual Registry Leaders Meeting in June of each year. The draft Annual Statistical Monitoring Report is discussed by the Committee, and approved by registries, before publication. Information available to the public observes data protection requirements concerning potential identification of individual cases.

### Analysis of cluster data in annual monitoring 2007–2013

The monitoring system was first run in 2003 for data up to birth year 2001, and significant adjustments in methodology were made until 2006. We report here the results of statistical monitoring run each year from 2007 to 2013.

According to whether they had been identified in the previous year’s monitoring, clusters were classified as “new”, “continuing” (increased in time and number of cases) or “old” (unchanged). “New” clusters may be in year X − 1 rather than in year X if the registry was not included in the monitoring analysis the previous year, if the statistical significance of findings shifted as the 5 year time period shifted, or if registration data for year X − 1 had been updated since the previous monitoring analysis.

Each scan test can identify more than one significant cluster [9], but for simplicity of presentation, we consider here only the most significant cluster from each scan test.

EUROCAT registries follow the ethics approval guidelines of their countries, and the EUROCAT Central Registry activities have ethics approval from Ulster University.

## Results

The annual results for the 7 years of annual monitoring from 2007 to 2013 (up to birth year 2011) are shown in Table 1. Across the 7 years, 165 (3.4 %) of all 4,823 cluster tests performed resulted in statistically significant clusters being detected. Of these 165, 126 (76 %) were “new”, 14 (8 %) were “continuing” and 25 (15 %) were “old” (Table 1). Ten of the 165 clusters were from date of birth-based tests. There were no instances of the same CA subgroup appearing as a cluster in a similar time period in two or more registries.

In the most recent year, 2013, 18 registries were included in monitoring covering an annual birth population of over 500,000 births (Table 1). In that 1 year, a total of 901 scan tests were performed, of which 3.8 % indicated a significant cluster, a total of 34 clusters.

Over the 7 year period, 126 “new” clusters were sent to registries for preliminary investigation. Of these, 9 % were not investigated by registries (Table 1), 21 % were considered to be diagnostically heterogeneous, 8 % were considered to be explained by changes in prenatal screening/diagnostic methods, and 28 % were considered to be explained by data quality issues (including duplicate cases, miscoding, changes in case ascertainment methods or inclusion criteria, or more accurate local data on LMP). For 35 % (44 clusters), the cluster was “confirmed” as an unusual aggregation. None of these clusters related to situations where there were any pre-existing environmental concerns in the registry area which might form a focus for investigation, nor did registry records identify any common exposures.

Most of the 44 confirmed new clusters involved 5–9 cases (Tables 2, 3). All four clusters with Observed/Expected ratio of 20 or more were of duration 1–2 days (Table 3). Five clusters were associated with a probability <0.001 (Tables 2, 3).

**Table 2:** Characteristics of 44 confirmed clusters

No. cases in cluster	
5–9 cases	35
10–19 cases	6
20+ cases	3
Duration of cluster in days	
7 days or less	8
8–14 days	1
15–30 days	7
31–60	7
61–180	11
181 days or more	10
Ratio of observed to expected cases	
<2	2
2–10	28
10–19	10
20+	4
*p* value	
<0.001	5
≥0.001 and <0.01	3
≥0.01 and <0.05	36

**Table 3:** Details of the 44 “confirmed” new clusters, 2007–2013

Congenital anomaly subgroup	Monitoring year	Observed cases in cluster (n)	Expected cases in cluster (n)	Observed/expected ratio	Duration of cluster (days)	Probability	Valid cases in CA subgroup over 5 years (n)	Trend detected
Spina Bifida	2007	5	0.38	13.2	9	0.049	59	No Significant change
Cleft lip with or without palate	2007	57	33.55	1.7	535	0.01	96	No Significant change
Congenital cataract	2008	6	0.43	14	30	<0.001	24	No Significant change
Ventricular septal defect	2008	6	0.32	18.8	1	0.045	502	Significant non-linear change
Ventricular septal defect	2008	18	4.6	3.9	50	0.012	140	Significant decreasing trend
Diaphragmatic hernia	2008	7	1.39	5	176	0.024	12	No significant change
Hip dislocation and/or dysplasia	2008	7	0.68	10.3	7	0.044	151	Significant non-linear change
Congenital constriction bands/amniotic band	2008	5	0.43	11.6	28	0.036	23	No significant change
Asplenia	2008	7	1.6	4.4	222	0.037	11	No significant change
Patau syndrome/trisomy 13	2008	7	0.72	9.7	54	<0.001	21	No significant change
Klinefelters syndrome	2008	17	6.87	2.5	455	0.013	23	No significant change
Anencephalus and similar	2009	5	0.27	18.5	21	0.005	20	Significant non-linear change
Coarctation of aorta	2009	9	2.08	4.3	243	0.009	13	Not tested: too few cases
Choanal atresia	2009	6	0.89	6.7	113	0.021	12	Not tested: too few cases
Cleft palate	2009	5	0.16	31.3	2	0.007	125	No significant change
Bladder exstrophy and/or epispadia	2009	5	0.92	5.4	174	0.039	8	Not tested: too few cases
Edward syndrome/trisomy 18	2009	9	1.76	5.1	86	0.039	31	No significant change
Neural tube defects	2010	5	0.25	20	2	0.048	193	No significant change
Atrioventricular septal defect	2010	5	0.58	8.6	62	0.039	14	Not tested: too few cases
Total anomalous pulm venous return	2010	5	0.62	8.1	87	0.028	11	Not tested: too few cases
Bilateral renal agenesis including Potter syndrome	2010	8	2.38	3.4	365	0.027	10	Not tested: too few cases
Renal dysplasia	2010	7	0.78	9	27	0.02	43	Significant non-linear change
Polydactyly	2010	54	30.56	1.8	532	<0.001	88	Significant decreasing trend
Patau syndrome/trisomy 13	2010	9	2.33	3.9	239	0.029	15	Not tested: too few cases
Hydrocephaly	2011	5	0.4	12.5	19	0.042	33	No Significant change
Down Syndrome	2011	5	0.15	33.3	1	0.01	233	Significant increasing trend
Anencephalus and similar	2012	9	1.32	6.8	27	0.024	73	No significant change
Transposition of great vessels	2012	5	0.47	10.6	34	0.039	21	No Significant change
Cleft lip with or without palate	2012	9	1.25	7.2	32	0.017	69	No significant change
Cleft lip with or without palate	2012	5	0.16	31.3	1	0.019	253	No significant change
Cleft palate	2012	7	0.93	7.5	64	0.019	23	No significant change
Omphalocele	2012	8	2.6	3.1	397	0.041	10	Not tested: too few cases
Renal dysplasia	2012	7	0.72	9.7	21	0.019	53	No significant change
Congenital hydronephrosis	2012	15	4.09	3.7	101	0.027	61	Significant non-linear change
Bladder exstrophy and/or epispadia	2012	7	1.17	6	197	<0.001	9	Not tested: too few cases
Club foot —talipes equinovarus	2012	11	1.94	5.7	37	0.018	79	No significant change
Down syndrome	2012	9	1.45	6.2	43	0.026	51	No significant change
Coarctation of aorta	2013	12	4.78	2.5	486	0.036	15	Not tested: too few cases
Total anomalous pulm venous return	2013	5	0.78	6.4	118	0.045	10	Not tested: too few cases
Ano-rectal atresia and stenosis	2013	5	0.82	6.1	139	0.039	9	Not tested: too few cases
Hypospadias	2013	37	15.86	2.3	102	0.024	232	Significant non-linear change
Club foot—talipes equinovarus	2013	6	0.56	10.7	5	0.049	144	Significant increasing trend
Patau syndrome/trisomy 13	2013	5	0.35	14.3	5	0.037	77	No significant change
Turner syndrome	2013	14	2.14	6.5	34	<0.001	92	No significant change

Of the 44 confirmed new clusters, 26 occurred wholly or partially within the most recent year of data (year X) with the registry recommending further monitoring rather than moving to a more extensive public health investigation. In the subsequent year of monitoring, 15 of these 26 reappeared as the same cluster, now classified as “old”, five were no longer statistically significant in the shifted 5 year period of the next year’s monitoring, and three were not re-evaluated as the registries concerned were not participating. Three of the 26 recent confirmed new clusters became “continuing clusters” in subsequent monitoring.

Eighteen of the 44 confirmed new clusters occurred in year (X − 1). For five of these clusters, the registry had not participated the previous year. Updating of year (X − 1) data with late registrations was the most probable explanation for the majority, but this could not be checked as timelines were only archived in case of significant clusters.

The scan test was usually picking up unusual aggregations not signalled by the trend analyses. Two of the 44 new confirmed clusters occurred against a background of a significant decreasing trend, and two occurred in association with an increasing trend and may have been a manifestation of that trend. For the remaining 40 clusters, either there was no significant trend (n = 22), or significant non-linear change (n = 6), or the numbers were too low for a trend test (n = 12) [Table 3].

Of the 14 continuing clusters detected in the 7 years of monitoring, 11 concerned situations where the original cluster had been explained by diagnostic, reporting or data quality issues, but it had not been possible to correct the data appropriately. Three were continuations of confirmed new clusters. One cluster of six cases of congenital cataract was first detected in 2008, with LMPs occurring in Feb–March of 2006, reappeared as an “old” cluster in 2009, and in 2010 increased in size by one case with LMP in April 2006. Due to the delay, no special attention was given to the continuation. One cluster of seven cases of asplenia was first detected in 2008, the registry did not participate in monitoring the next year, and in 2010 the cluster had grown to 11 cases. Again due to the delay no special attention was given. Finally, a cluster of six cases of clubfoot was detected in 2013 with LMPs in August 2010, had grown to 72 cases by 2014 with LMP from Sept 2009 to Feb 2011 (O/E 1.7, *p* < 0.001), and has been notified to the relevant national public health authority with as yet no further news.

## Discussion

During the 7 years of monitoring by EUROCAT 2007–2013, cluster detection revealed no evidence of a widespread, sudden and sustained temporal increase in CA potentially caused by a new teratogenic exposure. This cluster detection approach allows the existence of previously unsuspected or unrecorded exposures to be signalled, but is unlikely to detect the presence of teratogens of low potency [19], or teratogens with very restricted exposure such as medications for infrequent diseases or exposures in a small part of the geographical population. Other surveillance activities are needed in parallel, which tend to be more productive in terms of aetiologic findings. These include hypothesis generating screening of CA association with specific exposures or exposure sources (e.g. medications in pharmacovigilance [20], pollution sources or pollutants in envirovigilance) [21], and the testing of hypotheses with specifically designed studies based on the routinely collected registry data [22–24], and case–control surveillance investigating a wide range of potential aetiologic agents [24, 25].

We have not proceeded to detection of spatial clusters as this may be more appropriately done at regional or national level, and we consider spatial analyses of specific pollution sources [21, 26, 27] to be a more productive investment of resources than spatial cluster detection without prior hypothesis [28]. Moreover, fine spatial level analyses have more stringent data protection requirements which are difficult to achieve at European level currently.

Rothman advocated strongly [7] that cluster response should not be pursued as a public health activity, since most clusters would be chance unusual aggregations, and it is difficult to distinguish the few non-chance clusters and identify their causes, particularly when the number of cases is small. On the other hand, public health authorities are expected by the public to respond to suspected clusters with appropriate action [5, 6, 29]. Our experience suggests that a proactive approach to detecting and responding to clusters can be successful, rather than a reactive approach which responds to clusters identified by the public or media [6, 7]. A proactive approach has a number of advantages. The surveillance system has already established baseline frequency expectations and case ascertainment, which makes the verification of any unusual aggregation of cases relatively straightforward, a process which we also integrate in the data quality strategy [11]. Detecting clusters within a monitoring system avoids the silent multiple comparison problem where a large number of communities are implicitly monitoring their health but only the unusual aggregations are reported [5, 29, 30]. It avoids expending resources on following up suspected clusters which turn out not to be unusual aggregations [6]. It also avoids conducting an investigation in the difficult context of pressure from media or community groups who have identified a possible cluster. There have been no media or community concerns expressed about the 44 “confirmed” clusters we have detected, which are described in publicly available monitoring reports and other dissemination materials, and we believe that transparency is crucial.

Most registries or associated public health authorities, after confirmation of the cluster by preliminary investigation, decided on further monitoring rather than proceeding to full investigation. When further monitoring was done the next year, the vast majority of clusters did not increase in size, compatible with the clusters having been due to chance or a time-limited local cause. The “wait and see” approach may be a justifiable way to use public health resources most effectively, investing in continuing clusters which are least likely to be due to chance and where exposures, if real, are ongoing [31]. Nevertheless, the situation regarding public health response and triggering of full investigations [32] is variable and unclear in many European countries. Full investigation need not focus narrowly on the cluster, but investigate hypotheses in the wider population, so that scientific and public health benefit will result regardless of whether the cluster is finally fully explained [33, 34]. While we can be confident that the system has shown that no introduction of a widespread and highly teratogenic agent like thalidomide has occurred during this time period, we recommend that public health authorities in Europe develop clear protocols for response to CA clusters generated by routine statistical monitoring which have been confirmed by preliminary registry investigations.

We used expert review to help judge which clusters were less likely to be chance events. Similar clusters co-occurring in more than one registry would have been considered an indication of a non-chance event, as would more than one type of potentially aetiologically related CA clustering at the same time, but neither of these circumstances occurred. Continuing clusters also indicate a non-chance event. A key criterion for clinical geneticists was diagnostic homogeneity. In the early years of monitoring, many clusters were dismissed as heterogeneous, and this led to a better system for separating genetic syndromes in cluster detection. However, many known teratogens cause a number of different types of CA, and heterogeneous clusters with known exposures have been documented [35] so the future will lie in more aetiologically relevant classification of CA.

Another criterion was the size of the Observed/Expected ratio and associated *p* value. Neutra has suggested concentrating on clusters with a relative risk of 20 or more since any common cause, if present, will be most readily found by investigation [5]. The paradox, however, is that the higher the Observed/Expected ratio or RR, the more difficult it is to propose a plausible explanation [5]. In our experience, these very high ratios were associated with clusters of very short duration of 1 or 2 days which are unlikely to relate to plausible time-limited exposures. Moreover, day of conception was imprecisely estimated from gestational week, and the statistical method is being adjusted to reflect this.

The EMDP software enables registries to conduct more intensive early monitoring after a cluster is detected. The monitoring system can also be used reactively to speed up response to concerns expressed by clinicians—for example in 2006, a local concern about a seemingly unusual number of reduction defects of the hand led the registry concerned to intensify early data collection and monitoring of these defects in advance of the usual timetable, and request other registries to do so via EUROCAT Communications. This suspected case aggregation was finally found not to be unusual [36].

Use of the scan method which self-adjusts for multiple testing across multiple window sizes reduces the false positive rate, and the number of clusters detected was not overwhelming. Data simulations showed that, since only clusters in the most recent two of the 5 year period are output, approximately 2.5 % of tests would detect eligible clusters by chance [9]. The actual “detection rate” (i.e. proportion of tests resulting in statistically significant temporal clusters of cases) was 3.4 %, but only one-third were confirmed as unusual aggregations, bringing the detection rate below the chance detection rate. Cluster investigation concentrates on the cases “inside” the cluster, but not those “outside”—only cases within the cluster are excluded due to data errors, thus clusters disappear but cannot appear as a result of verification, leading to a lower than chance detection rate of confirmed clusters. Another issue may be a desire to explain a cluster as an artefact of diagnostic or other change rather than be left with an unexplained excess. Registry Leaders Meetings and expert review are important for discussing these judgements.

Timeliness is one of the major issues we face. The sensitivity of the cluster detection method to data completeness was shown by the high proportion of clusters (up to 30 % of confirmed new clusters) which were missed at the first monitoring opportunity, and only found when data had been further updated with late or amended diagnoses. We have found that earlier data transmission is not feasible and have instead increased efficiency of the statistical monitoring process. While monitoring across healthcare databases in real time is becoming technically feasible in some areas, speed would come at the expense of noise relating to poorly standardised diagnosis and coding of CA in healthcare data, missing and inaccurate data, and absence of information about terminations of pregnancy, thus increasing the false positive rate and reducing the sensitivity of identification of clusters of concern.

EUROCAT’s statistical monitoring covers 10 % of the EU population, less than half of EUROCAT’s current surveillance coverage. There is thus potential, particularly with use of the EDMP by registries locally, to increase population coverage.

We conclude that monitoring for CA clusters based on date of conception is a useful and feasible part of surveillance, particularly in relation to early detection of widespread exposures with high teratogenicity, accompanied by clear investigation protocols and expert review. The results for 2007–2013 have been reassuring in terms of the absence of widespread, sudden and sustained temporal increase in CA potentially caused by a new teratogenic exposure. Temporal cluster detection is only one of the various types of surveillance based on birth defect registries needed to detect teratogenic exposures in the population, and one of the most limited in terms of progressing aetiologic understanding. However, when integrated with a data quality improvement strategy, temporal cluster detection need not be resource intensive and is a useful public health tool, whether the results are negative or positive in terms of finding a cluster of concern. The greatest improvements to the temporal cluster detection system would be greater timeliness of data availability, greater clarity in local public health response protocols, development of a more aetiologically-oriented CA classification system, and expansion of the participating European population.
